# Intergenerational patterns of mental health problems: the role of childhood peer status position

**DOI:** 10.1186/s12888-019-2278-1

**Published:** 2019-09-18

**Authors:** Evelina Landstedt, Ylva B. Almquist

**Affiliations:** 1 Department of Public Health and Clinical Medicine, Epidemiology and Global Health, Umeå University, Norrland University Hospital, SE-901 85 Umeå, Sweden; 2 Department of Public Health Sciences, Centre for Health Equity Studies (CHESS), Stockholm University, SE-106 91 Stockholm, Sweden

**Keywords:** Birth cohort, Intergenerational patterning, Longitudinal, Mental health, Peer relationships, Sweden

## Abstract

**Background:**

Past research has established the intergenerational patterning of mental health: children whose parents have mental health problems are more likely to present with similar problems themselves. However, there is limited knowledge about the extent to which factors related to the child’s own social context, such as peer relationships, matter for this patterning. The aim of the current study was to examine the role of childhood peer status positions for the association in mental health across two generations.

**Methods:**

The data were drawn from a prospective cohort study of 14,608 children born in 1953, followed up until 2016, and their parents. Gender-specific logistic regression analysis was applied. Firstly, we examined the associations between parental mental health problems and childhood peer status, respectively, and the children’s mental health problems in adulthood. Secondly, the variation in the intergenerational patterning of mental health according to peer status position was investigated.

**Results:**

The results showed that children whose parents had mental health problems were around twice as likely to present with mental health problems in adulthood. Moreover, lower peer status position in childhood was associated with increased odds of mental health problems. Higher peer status appeared to mitigate the intergenerational association in mental health problems among men. For women, a u-shaped was found, indicating that the association was stronger in both the lower and upper ends of the peer status hierarchy.

**Conclusions:**

This study has shown that there is a clear patterning in mental health problems across generations, and that the child generation’s peer status positions matter for this patterning. The findings also point to the importance of addressing gender differences in these associations.

## Background

Researchers have long been interested in the intergenerational transmission of social disadvantages and health [1–5], including intergenerational patterns of mental health [1–5]. As a result, the association between parents’ mental health and their children’s risk of psychological distress and internalising symptoms, depression and anxiety, bipolar disorders, and post-traumatic stress syndrome is well established [6–13]. Furthermore, existing research, albeit limited, suggests that maternal intergenerational transmission of mental health problems is particularly strong [13–15], and especially so among girls [8, 16]. It should, nevertheless, be noted that the majority of studies using self-reported data on parental mental health status has relied on reports by mothers only [13, 17, 18]. Also, the above-mentioned studies have primarily focused on the child generation’s mental health in childhood or adolescence and have to a lesser extent examined mental health outcomes across the entire span of adulthood.

The mechanisms behind the intergenerational patterns of mental health reflect a complex web of genetic and environmental factors [11, 14, 19]. Although we acknowledge that gene-environment interaction explanations are highly plausible, the present study focuses on the role of social (environmental) circumstances. This approach is supported by previous research concluding that the intergenerational transmission of poor mental health cannot solely be explained by genetic factors and that environmental influences are salient [14, 15, 20]. Typically, environmental circumstances include family factors such as conflicts and parenting [17] or, in studies looking at mental health outcomes in adulthood, the social reproduction of educational pathways and occupational choices [2, 21]. The approach of health selection implies that people with poor mental health are assumed to be found in the lower end of the social ladder [22]. Others argue that health selection fails to provide a complete picture since life circumstances in childhood (reflecting conditions related to the parents) are crucial to later mental health. Hence, social causation would precede health selection [2]. However, both the health selection and the social causation hypotheses focus on social circumstances of the parental generation. The importance of the child’s experience of social contexts outside the family, such as social relationships with peers, has received little attention in this line of research.

Peer status has been shown to be a key component of childhood social relationships; in all peer networks, such as the school class, a hierarchical structure of status evolves over time [23, 24]. Lower peer status has consistently been linked to increased risks of a wide range of adverse outcomes, including mental health problems [25–28]. The mechanisms underlying this association include, for example, exposure to peer victimization and bullying which negatively affects the individual’s sense of security and self-worth [29, 30]. Conversely, higher peer status is linked to better mental health outcomes through, for example, the provision of various types of support gained by having close relationships and holding a more central position in the peer network. It also involves more general resources that can influence health, such as access to information as well as power and influence [25, 31–33].

Drawing on cumulative (dis) advantage theory [34, 35], peer status can be seen as part of a broader clustering of risk – or protective – factors in childhood, that give rise to a gradual increase of disadvantages – or advantages – over the life span. As such, influences of the child’s peer status position on their mental health may be extended into adulthood. Nevertheless, having mentally ill parents does not automatically lead to lower peer status: most of these children will still be able to form positive relationships with peers and obtain high positions in the peer status hierarchy. Previous studies show, for example, that childhood peer status is associated with mental health in adulthood, independently of parental mental health problems [36]. Moreover, a vast amount of studies demonstrate that positive social relationships can mitigate the influences of negative life events and conditions on mental health [37]. However, to the best of our knowledge, no studies have examined the potentially buffering role of peer status for the intergenerational transmission of mental health problems. We hypothesise that low peer status will reinforce the intergenerational transmission of poor mental health whereas high peer status will buffer against later mental health problems and more so among those from families with no parental poor mental health.

As noted, research has suggested that the strength of the intergenerational transmission of mental health problems differs depending on the gender of both the parent and the child [8, 13–16]. Findings on gender differences concerning the association between peer status and mental health are nevertheless inconsistent [27, 36, 38]. For example, a Scottish cross-sectional study on the link between well-being and three dimensions of school-based subjective social status revealed no gender differences [38], while Swedish longitudinal findings showed an increased risk of adult anxiety and/or depression for women, but not for men, who held low childhood peer status positions [36]. Hence, more is yet to be learned about the role of gender in both the mental health transmission between generations and the peer status – mental health associations.

In sum, there are three main gaps in current research on the intergenerational patterns of mental health problems. First, most research focuses on child or youth, and not adult, mental health in the child generation. A comprehensive intergenerational life course analysis requires a longer temporal perspective [39]. Second, there is a lack of studies that have highlighted the variation in the strength of the intergenerational transmission of mental health and, in particular, the importance of environmental factors related to the child’s own social contexts, such as peer status position. Third, more analyses on gender patterns are needed. In contrast to previous studies which have relied on self-reported data from parents which in most cases equal mothers, our study has data for both parents and, moreover, examines the maternal and or paternal links separately for boys and girls.

The present study will address the above-specified knowledge gaps, as formulated in the following research questions:

1. To what extent are parental mental health problems associated with the child generation’s mental health problems in adulthood?
2. In what way does this pattern vary according to the child generation’s peer status position in childhood?
3. Are there gender differences in any of the above-mentioned associations?

## Methods

### Data material

Data were drawn from the Stockholm Birth Cohort Multigenerational Study (SBC Multigen), which was created in 2018/2019 through a probability matching of two anonymous datasets. The first dataset was the Stockholm Metropolitan Study (SMS), encompassing all individuals born in 1953 who resided in the greater Stockholm metropolitan area in 1963 (*n* = 15,117). The second was RELINK53, defined as all individuals who were born in 1953 and resident in Sweden in 1960, 1965, and/or 1968, as well as their ascendant, contemporaneous, and descendant family members (*n* = 2,390,753). By using a matching algorithm based on 21 variables identical to both datasets, 14,608 could be positively matched and thus included in the SBC Multigen. These individuals constitute our ‘child generation’, whereas their mother and fathers are referred to below as the ‘parental generation’.

### Variables

Information about mental health problems in the parental generation was derived from the Social Register (part of the SMS), covering the period 1953 to 1972 (i.e. from the birth of the child generation until age 19). The Social Register contains records kept by the family and child welfare services, manually collected from the municipalities in the Stockholm region. The original data contained information about whether the father/mother had psychiatric problems or suffered from depression, received psychiatric treatment, or committed suicide. For the purpose of the current study, all of the above were seen as indicative of mental health problems.

Concerning mental health problems in the child generation, data were derived from the National Patient Register (part of RELINK53), covering the period 1973 to 2016 (i.e. when the child generation was aged 20 to 63). This data refers to diagnoses related to overnight stays at the hospital, i.e. in-patient care (including psychiatric care). The National Patient Register has more or less full coverage for hospitals in the county of Stockholm from 1973 and onwards. Based on the International Classification of Diseases (ICD), diagnoses from the chapter ‘Mental disorders’ according to the 8th and 9th revisions as well as from the chapter ‘Mental and behavioural disorders’ according to the 10th revision were seen as indicative of mental health problems. However, cases of hospitalization due to substance use disorders (since these rather reflect behavioural types of disorder) or mental retardation and diseases originating in childhood (since they are likely to precede the measure of peer status) were not included (ICD 8: 303–304, 308–315; ICD 9: 303–305, 312–319; ICD 10: F10-F19, F70-F98). Among the individuals included in the SBC Multigen, 7.6% (*n* = 1101) individuals had been hospitalised due to mental health problems. The most common diagnoses were schizophrenia, depressive disorders, and bipolar affective disorders.

Information about the child generation’s peer status position was based a sociometric test in 1966 (age 13), which was part of the so-called School Study (part of the SMS). Whereas the School Study was included all cohort members who attended schools in the Stockholm metropolitan area, the sociometric test was only administered to cohort members who were in 6th grade. Children were asked to nominate three classmates with whom they preferred to work with in class. As discussed and confirmed by previous studies, this question reflects the extent to which the individual is accepted and liked by their peers [40]. Based on the number of received nominations, the following categories were derived: ‘Marginalised’ (0 nominations), ‘Low status’ (1 nomination), ‘Medium status’ (2–3 nominations), and ‘High status’ (4 or more nominations).

Control variables included school class size (1966) and social class in the parental generation (based on the occupation of the head of the household in 1953), both drawn from the SMS.

For the distribution of all study variables, see Table 1.

**Table 1:** Descriptive statistics of the study variables (*n* = 12,120)

	Men *n* = 5998		Women *n* = 6122		% Mental health problems in the parental generation		% Mental health problems in the child generation	
	N	%	n	%	Men	Women	Men	Women
Mental health problems in the parental generation								
No	5645	94.1	5774	94.3	–	–	6.4	6.9
Yes	353	5.9	348	5.7	–	–	11.9	14.1
Mental health problems in the child generation								
No	5595	93.3	5675	92.7	5.6	5.3	–	–
Yes	403	6.7	447	7.3	10.4	11.0	–	–
Peer status position								
Marginalised	777	13.0	582	9.5	9.1	9.1	8.0	9.8
Low status	1165	19.4	1298	21.2	7.8	7.5	8.5	8.5
Medium status	2126	35.5	2398	39.2	5.6	5.3	6.6	6.8
High status	1930	32.2	1844	30.1	3.7	3.8	5.2	6.3
Class size	Range: 10–37, Median: 27		Range: 10–40, Median: 27		–	–	–	–
Social class in the parental generation								
Upper/middle class	3108	51.8	3311	50.8	4.3	4.4	7.0	6.9
Working class	2726	45.5	2839	46.4	7.6	7.0	6.4	7.5
Unclassified	164	2.7	172	2.8	6.7	5.8	7.3	11.1

### Statistical analysis

Only individuals with complete information for all study variables were included in the analysis (*n* = 12,120). The attrition was due to a) migration out of the Stockholm metropolitan area before the School study of 1966, and b) non-participation in the sociometric test. In the latter case, it should again be noted that only cohort members attending 6th grade where invited to take part in the test, which thus excluded all individuals who had advanced or been held back one or more grades. Furthermore, we restricted the sample to individuals who attended classes with ten or more individuals since the peer status distribution is somewhat truncated in smaller school classes.

Gender-specific logistic regression analysis (producing odds ratios; OR) was used to analyse the extent to which parental mental health problems are associated with subsequent mental health problems in the next generation of (adult) children, and whether peer status position is related to adult mental health problems in the child generation. Two models were generated, of which the first (Model 1) was adjusted for school class size and social class in the parental generation, whereas the second (Model 2) additionally included mutual adjustment for mental health problems in the parental generation and peer status position in the child generation.

We also constructed an eight-category variable based on each combination of mental health problems in the parental generation and peer status position in the child generation. This variable was used in gender-specific logistic regression analyses to examine in what way the intergenerational association in mental health problems varied according to the child generation’s peer status position. As a formal test for interaction, we derived model fit statistics for a) a model that included mental health problems in the parental generation and peer status position in the child generation, with b) a model that also included the (dummies of the) combination variable. To further illustrate the interaction between mental health problems in the parental generation and peer status position in the child generation, the *adjprop* module in Stata was applied, through which it was possible to calculate adjusted probabilities and confidence intervals (expressed as percentages) from the logistic regression estimates.

As described above, the analyses were adjusted for school class size. To further account for the potentially differential distribution (and meaning) of peer status across school classes of varying sizes, we re-analysed the results using multilevel mixed-effects logistic regression modelling with adjustment for class size at the school class level. The associations between parental mental health problems and own mental health problems in adulthood on the one hand, and between peer status position and own mental health problems on the other hand, remained largely the same, although the estimates were slightly reduced (data not presented).

## Results

Table 2 demonstrates the extent to which mental health problems in the parental generation and peer status position are associated with mental health problems in the child generation. The results from Model 1 show, firstly, that men and women whose parents suffered from mental health problems are around twice as likely to experience such problems themselves (men: OR = 1.97; women: OR = 2.16, respectively). These estimates are slightly reduced when peer status position is included in Model 2, but remain statistically significant. Secondly, with regard to the association between childhood peer status position and later mental health problems among men, the results from Model 1 show that those in medium-status positions have increased odds (OR = 1.29) compared to those with high status, although this estimate does not reach a statistically significant level. Men in low-status and marginalised positions demonstrate larger and significant estimates: OR = 1.70 and OR = 1.60, respectively. The corresponding results for women in Model 1 looks similar, with a clearer peer status gradient in mental health problems: here, those in medium-status positions have increased (statistically non-significant) odds (OR = 1.07) for mental health problems compared to women in high-status positions, whereas women in low-status and marginalised positions show estimates of OR = 1.35 and OR = 1.57, respectively. For men and women alike, only marginal changes occur in Model 2, when mental health problems in the parental generation are included.

**Table 2:** Odds ratios (OR) of mental health problems in the child generation, as predicted by the parental generation’s mental health problems and the child generation’s peer status position. Results from logistic regression analysis (*n* = 12,120)

	Mental health problems in the child generation OR (95% Confidence Intervals)			
	Men (*n* = 5998)		Women (*n* = 6122)	
	Model 1^a^	Model 2^b^	Model 1^a^	Model 2^b^
Mental health problems in the parental generation				
No (ref.)	1.00	1.00	1.00	1.00
Yes	1.97 (1.40, 2.77)	1.86 (1.32, 2.62)	2.16 (1.57, 2.98)	2.08 (1.51, 2.87)
Peer status position				
Marginalised	1.60 (1.15, 2.22)	1.53 (1.11, 2.14)	1.57 (1.13, 2.19)	1.50 (1.07, 2.10)
Low status	1.70 (1.28, 2.27)	1.65 (1.24, 2.21)	1.35 (1.03, 1.77)	1.31 (1.00, 1.72)
Medium status	1.29 (0.99, 1.68)	1.27 (0.98, 1.66)	1.07 (0.84, 1.37)	1.05 (0.82, 1.35)
High status (ref.)	1.00	1.00	1.00	1.00

^a^ Adjusted for school class size and social class in the parental generation

^b^ Adjusted for school class size and social class in the parental generation + adjusted for mental health problems in the parental generation and peer status position, respectively

Table 3 concerns the combinations of the parental generation’s mental health problems and the child generation’s peer status position in relation to mental health problems in the child generation. Overall, men and women who hold high-status positions and whose parents do not have mental health problems have the lowest odds of having own mental health problems. In comparison to this group, those in marginalised positions whose parents have mental health problems have more than a three-fold odds of subsequent mental health problems (men: OR = 3.84; women: OR = 3.13). The remaining combinations demonstrate odds ratios ranging somewhere in between. Here, a quite clear gradient is shown for men (with the exception of men in marginalised and low status positions whose parents did not have mental health problems). Among women, the combinations of having parents with mental health problems and either medium or high status among women show higher odds than what may have been expected, at the same time as the combination of parental mental health problems and low status demonstrates an unexpectedly low odds ratio.

**Table 3:** Odds ratios (OR) of mental health problems in the child generation, as predicted by combinations of the parental generation’s mental health problems and the child generation’s peer status position. Results from logistic regression analysis (*n* = 12,120)

	Mental health problems in the child generation OR (95% Confidence Intervals)	
	Men (*n* = 5998)	Women (*n* = 6122)
	Model 1^a^	Model 1^a^
Mental health problems in the parental generation + Peer status position		
Yes + Marginalised	3.84 (1.99, 7.42)	3.13 (1.49, 6.60)
Yes + Low status	3.11 (1.67, 5.81)	1.77 (0.90, 3.52)
Yes + Medium status	1.88 (0.97, 3.62)	2.67 (1.58, 4.52)
Yes + High status	1.70 (0.72, 4.04)	2.85 (1.45, 5.59)
No + Marginalised	1.44 (1.01, 2.06)	1.55 (1.08, 2.21)
No + Low status	1.64 (1.21, 2.22)	1.42 (1.07, 1.88)
No + Medium status	1.29 (0.98, 1.70)	1.06 (0.82, 1.37)
No + High status (ref.)	1.00	1.00

^a^ Adjusted for school class size and social class in the parental generation

Interaction analyses were additionally performed to confirm that the parental generation’s mental health problems and the child generation’s peer status position interacted in their association with subsequent mental health problems in the child generation. Drawing on model fit assessments (based on the Akaike Information Criterion; AIC), it can be concluded that the model that included the interaction term provided a slightly better fit to data compared to the model without the interaction term, for men but not for women (Men: AIC = 2947.133 vs. AIC = 2944.136; Women: AIC = 3185.223 vs. AIC = 3185.391).

**Fig. 1:**
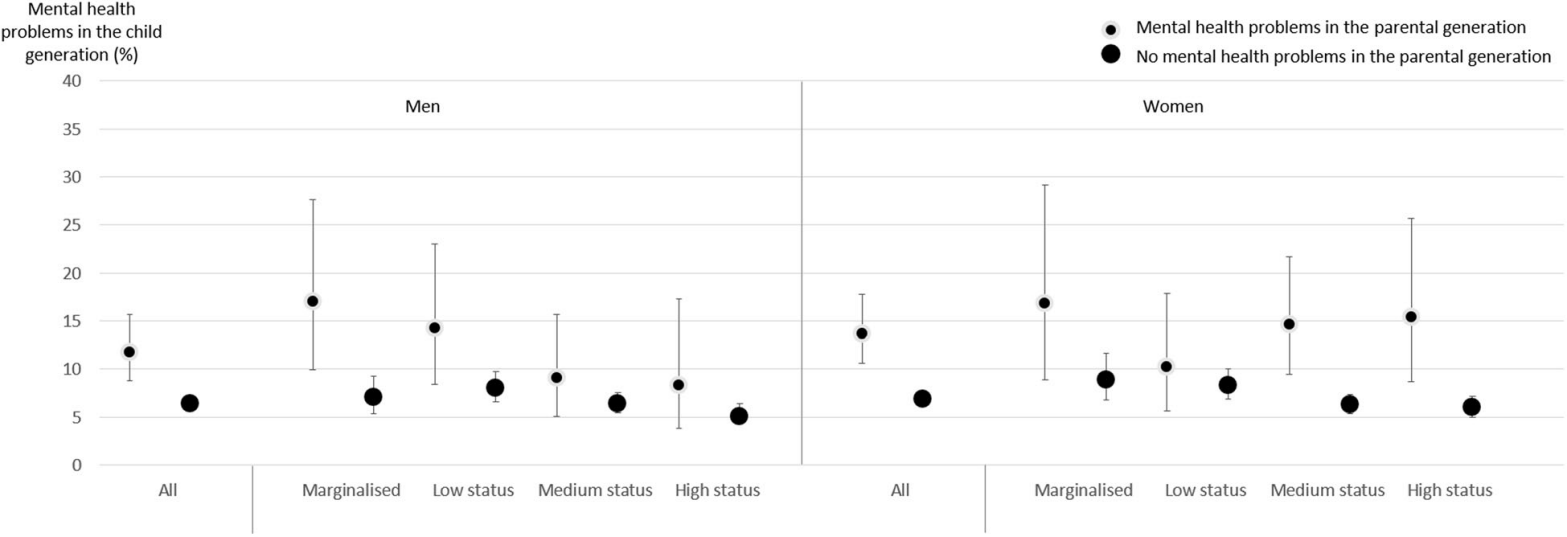
Proportions (expressed as percentages, with 95% confidence intervals) of mental health problems in the child generation, as predicted by the parental generation’s mental health problems in combination with the child generation’s peer status position (Men: *n* = 5998; Women: 6122). Adjusted for school class size and social class in the parental generation

Figure 1 shows proportions of mental health problems in the child generation as predicted by the parental generation’s mental health problems. Starting with the results for men, the left-hand side of the table demonstrates that among those whose parents had mental health problems, 11.8% end up having the similar types of problems. The corresponding percentage among those whose parents did not have mental and behavioural problems is 6.4. When examining the variation across peer status position, the results vary substantially. For example, among men whose parents had mental health problems, 17% of those in marginalised positions and 14.2% of those with low status, end up having mental health problems in adulthood. The corresponding numbers for men with medium and high status are 9.1 and 8.3%, respectively. Among men whose parents did not have mental health problems, the differences across status positions are less pronounced, ranging from 5.1 to 8%.

The right-hand side of the figure shows the results for women. Overall, among those whose parents had mental health problems, 13.7% end up having mental health problems themselves. The corresponding percentage among those whose parents did not have mental health problems is 6.9. Similar to men, the proportion of own mental health problems is largest among marginalised women whose parent had mental health problems (16.8%). However, in contrast to the results for men, there is no evidence of a gradient across the positions among those whose parents had mental health problems: women with medium and high status have unexpectedly high levels of own mental health problems: 14.6 and 15.4%, respectively, whereas the percentage for women in low status positions is 10.2. Focusing on women whose parents did not have mental health problems, there is a clear gradient in own problems across the status positions, ranging from 8.9% among the marginalised to 6% among those with high status.

Overall, the results are rather different for men and women. For men, an expected ‘dose-response’ pattern was identified, whereas for women, those presenting with a combination of parental mental health problems and marginalised, medium, or high childhood peer status were at particular risk of adult mental health problems.

As a sensitivity analysis, we repeated Fig. 1 for fathers and mothers, respectively. Additional file 1: Figure S1 shows the proportion of mental health problems in the child generation as predicted by the father’s mental health problems, whereas Additional file 2: Figure S2 illustrates the corresponding results for mothers’ mental health problems (see Additional files 1 and 2). Overall, the results are similar. However, there is a tendency for men in marginalised peer status positions to show higher proportions of own mental health problems if the subsequent generation’s mental health problems were present among the fathers in comparison to the mothers. This finding is the opposite among men with low status. For women, slightly higher levels of own mental health problems are found if the mother was the one with mental health problems. The shape of the pattern across peer status positions nevertheless seems to be independent of the parental gender.

## Discussion

The present study, based on a large Swedish cohort born in 1953 and their parents, shows that individuals whose parents suffered from mental health problems are more likely to also present with poor mental health in adulthood as measured by any hospital admission due to a number of psychiatric diagnoses, for example depressive disorders and bipolar affective disorders. These findings confirm existing research of intergenerational transmission of mental health [6–12]. However, this study contributes to the literature through its focus on adult mental health, in contrast to outcomes in childhood or adolescence. Another important difference compared to previous research is the low risk of response bias due to the use of register data instead of self-reported measures of mental health.

The main focus of this study was to explore whether intergenerational patterns of mental health differed depending on social/environmental circumstances in the child generation’s childhood years. It is well established that supportive social relationships are protective against mental health problems, whereas loneliness, weak peer networks, and peer problems (such as bullying) in childhood are risk factors of the same, both in childhood/adolescence [26, 41] and longitudinally into adulthood [36, 38]. The present study contributes with new knowledge through acknowledging the conjunction of both parental and child generation factors. As hypothesised, the identified intergenerational transmission of poor mental health was most evident among those in lower peer status positions at age 13. This suggests that childhood peer networks play an important role in the ‘spill-over effect’ of parental health [5].

A linear patterning of adult mental health problems depending on parental mental health and own childhood peer status was confirmed for men. In other words, the lower the peer status position of sons of parents with mental health problems, the greater the likelihood of presenting with own mental health problems in adulthood. Several possible mechanisms could explain why childhood peer status influences the strength of the intergenerational transmission of mental health problems. For example, children of mentally ill parents are not only at risk of poor attachment and neglect due to conflicts and inadequate parenting [17], they may also struggle with social stigma and shame of having a mentally ill parent which places those children in vulnerable positions in relation to peer interaction and making friends [42]. It is also possible that boys to a higher degree than girls display less prosocial behaviour as a reaction to their parents’ mental health problems and thereby experience more trouble with friends, including bullying [18, 43].

Furthermore, previous research suggests that boys and men generally have fewer close friends and weaker social networks than girls and women [24, 44]. Given the strong mental health protective effects of social relationships, boys with low peer status from families with mental health problems may be particularly sensitive and at risk of developing own poor mental health. Typically, this pattern would also apply to girls. However, the findings suggest otherwise. Among women, the pattern was u-shaped; the combination of parental mental health problems and either very low (marginalised) or medium/high peer status increased the likelihood of adult mental health problems. These findings merit further attention. It is possible that gendered norms and expectations with regards to popularity and status play a role here. Studies suggest that aggressiveness is dominant in the perception of popularity in boys [45], while prosocial behaviour is valued in relation to popularity in girls [23, 46]. Hence, it is possible that for girls, being popular implies increased social pressure that might take its toll on their mental health through relational stress. Stress from social and relational responsibilities has been shown to be more prevalent as well as more detrimental for mental health in girls than in boys [46, 47].

To rule out the likely influence of childhood mental health status on both peer status and later mental health, analyses were adjusted for internalizing and externalizing problems in the child generation (data not shown). In accordance with Shepman [13], this did not substantially alter the results. In other words, childhood peer status matters for mental health in adulthood, especially for those with mentally ill parents, regardless of the child’s own mental health status.

Our findings did not show any clear differences in the strength of intergenerational transmission of mental health problems based on the gender of the parent. In the few existing studies exploring both maternal and paternal transmission of mental health, the associations seem stronger for same-gender-dyads than mother-son/father-daughter dyads [8, 16]. Andreas and colleagues [16] suggest that gendered parenting styles or gender cognition theory may explain such findings. Importantly, these hypotheses also suggest reciprocity, i.e., if the child identify with the parent of the same gender, this also applies to the parent. It is possible that this influences how parents report the mental health status of their child, a procedure applied in many studies [18]. Our results may contradict previous findings because mental health data for both parents and children were drawn from registers instead of self-reports.

In line with previous studies, the current study has shown that positive peer relationships, as manifested through higher peer status positions, are overall beneficial for subsequent mental health outcomes. From a policy perspective, the results imply that school-based interventions aimed at improving children’s experiences with peers would provide an opening for also improving healthy development. This appears to apply particularly to boys. Important to note, however, is the possibility that girls how have mentally ill parents may come to respond differently to such efforts, at least in cases where more intensive peer interaction is accompanied by increases in social pressure and relational stress.

### Strengths and limitations

The major strength of this study is that it builds on prospective data collected for a relatively large, community-based sample. Some limitations should nevertheless be addressed. One apparent shortcoming is that we have no access to genetic information which makes it impossible to determine if the intergenerational association in mental health problems is reliant upon the inheritance of genes related to mental illness or whether it is due to the assumingly troublesome circumstances that children living with mentally ill parents are exposed to. However, it should be emphasised that the primary focus of the current study was not to draw any causal inferences but, rather, whether the association varied according to experiences related to the children’s own social contexts, such as those reflected through peer status position. The empirical analysis did furthermore not set out to *explain* the associations between parental mental health problems, childhood peer status position, and own mental health problems in adulthood; therefore, we restricted the set of covariates to include only the most important confounders. An examination of potential pathways would require a careful consideration of temporality as well as appropriate statistical tools for assessing mediation, such as structural equation modelling. These are issues for future studies to look further into.

Another limitation concerns the measurement of mental health problems. For the child generation, we have relied on in-patient care, which captures only the most severe cases. Some other important, and potentially conflicting, sources of bias may also be present here. On the one hand, children whose parents’ mental health problems are registered by the authorities may be more closely monitored by the social and health care services and thus be more likely become diagnosed with a mental illness. On the other hand, help-seeking behaviours may be positively associated with peer status, which would lead individuals with lower status positions to become diagnosed to a lesser extent than expected. Moreover, due to the small proportion of individuals being hospitalised due to mental disorders, we were not able to examine specific diagnoses (e.g. depression and/or anxiety) which potentially could have provided deeper insights into possible mechanisms behind the patterning found here.

For the parental generation, only a very broad indicator of mental health problems was available in our data. A related issue has to do with the generalisability of the results when it comes to later cohorts. For example, having a mentally ill parent may have meant something different in our 1953 cohort compared to children of today: increased awareness in the general population around mental health problems could suggest that the stigma surrounding this topic has decreased since the 1950s and 1960s and, to the extent to which the intergenerational association in mental health problems is socially induced, this would lead to a weakening of the intergenerational transmission over time.

Finally, we only had information about peer status position at age 13. It would have been preferable to have assessments of peer status across multiple time points in order to account for stability and change.

## Conclusions

This study shows that there is a clear patterning in mental health problems across generations, and that the child generation’s peer status positions matter for this association. Presenting with own mental health problems in adulthood was particularly evident among men who were located in the lower end of the status distribution in childhood. For women with mentally ill parents, also those with high peer status seemed to be at increased risk of subsequent mental health problems. Overall, the findings point to the relevance of focusing on childhood social contexts outside that of the family for understanding the transmission of mental health.

## Supplementary information

**Additional file 1: Figure S1.** Proportions (expressed as percentages, with 95% confidence intervals) of mental health problems in the child generation, as predicted by the parental generation’s mental health problems (only fathers) in combination with the child generation’s peer status position (Men: *n* = 5998; Women: 6122). Adjusted for school class size and social class in the parental generation.

**Additional file 2: Figure S2.** Proportions (expressed as percentages, with 95% confidence intervals) of mental health problems in the child generation, as predicted by the parental generation’s mental health problems (only mothers) in combination with the child generation’s peer status position (Men: *n* = 5998; Women: 6122). Adjusted for school class size and social class in the parental generation.

## Data Availability

The datasets generated and/or analysed during the current study are not publicly available due ethical regulations regarding the Stockholm Birth Cohort Multigenerational Study (SBC Multigen) but are available from the corresponding author on reasonable request.

## Abbreviations

AIC: Akaike Information Criterion
CI: Confidence intervals
ICD: International Classification of Diseases
OR: Odds ratio
RELINK: Reproduction of inequality through linked lives
SBC: Stockholm Birth Cohort
SMS: Stockholm Metropolitan Study
