# Supplementary figures and images for: Intergenerational patterns of mental health problems: the role of childhood peer status position
Source: BMC Psychiatry. 2019 Sep 18;19:286. doi: 10.1186/s12888-019-2278-1 (PMC6749655; doi:10.1186/s12888-019-2278-1)

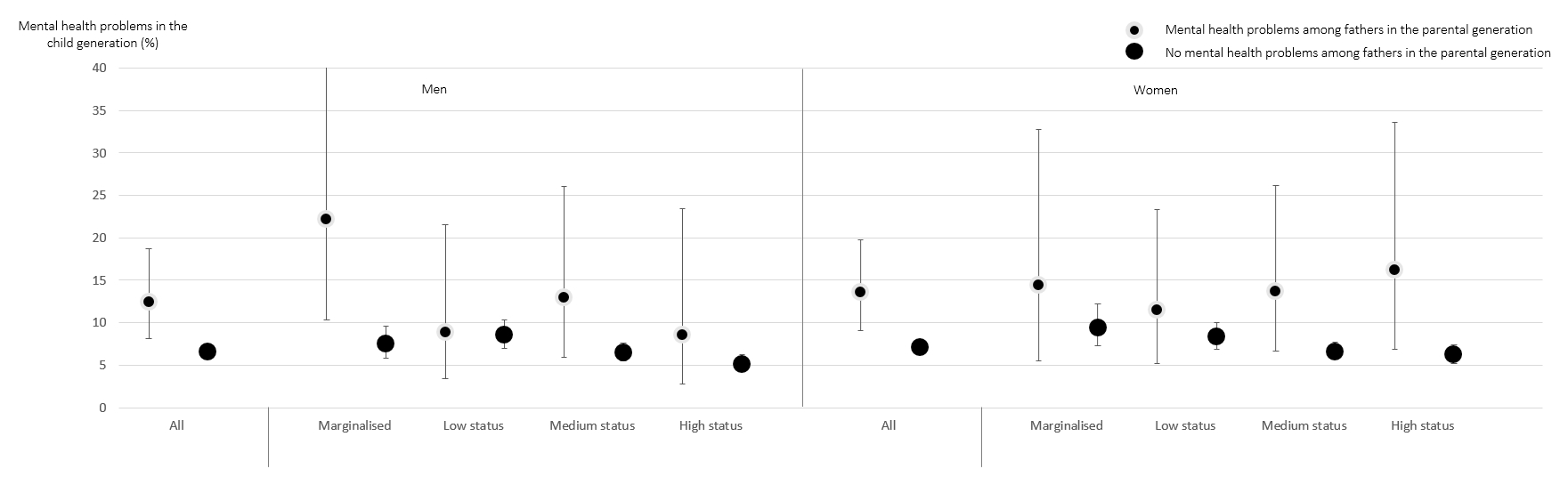

Supplement: Supplementary file 1 — Additional file 1: Figure S1. Proportions (expressed as percentages, with 95% confidence intervals) of mental health problems in the child generation, as predicted by the parental generation’s mental health problems (only fathers) in combination with the child generation’s peer status position (Men: n = 5998; Women: 6122). Adjusted for school class size and social class in the parental generation. [file 12888_2019_2278_MOESM1_ESM.jpg]

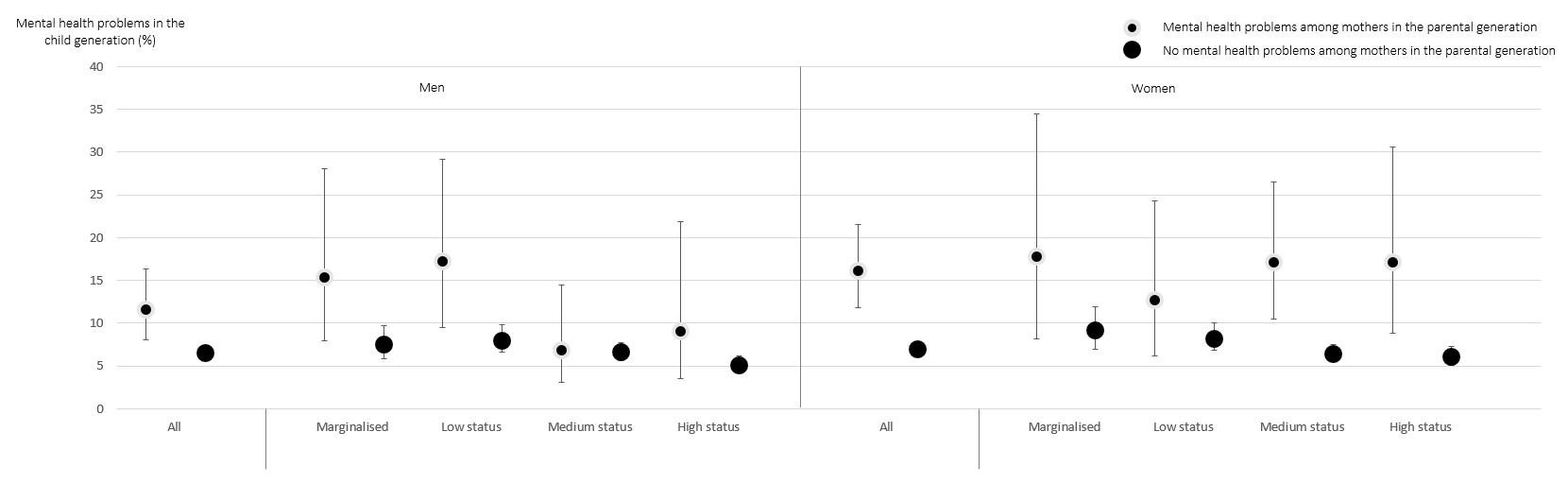

Supplement: Supplementary file 2 — Additional file 2: Figure S2. Proportions (expressed as percentages, with 95% confidence intervals) of mental health problems in the child generation, as predicted by the parental generation’s mental health problems (only mothers) in combination with the child generation’s peer status position (Men: n = 5998; Women: 6122). Adjusted for school class size and social class in the parental generation. [file 12888_2019_2278_MOESM2_ESM.jpg]
